# Plants and mental disorders: the case of Catalan linguistic area

**DOI:** 10.3389/fphar.2023.1256225

**Published:** 2023-10-31

**Authors:** Laia N. Irún, Airy Gras, Montse Parada, Teresa Garnatje

**Affiliations:** ^1^ Institut Botànic de Barcelona (IBB), CSIC-Ajuntament de Barcelona, Barcelona, Spain; ^2^ Laboratori de Botànica—Unitat Associada CSIC, Facultat de Farmàcia i Ciències de l’Alimentació—Institut de Recerca de la Biodiversitat—IRBio, Universitat de Barcelona, Barcelona, Spain

**Keywords:** Catalan linguistic area, ethnobotany, Iberian Peninsula, medicinal plants, mental disorders, mental health, traditional knowledge

## Abstract

**Introduction:** Mental disorders are among the leading causes of ill-health and disability worldwide. Despite the disease burden they cause, including significant direct and indirect impacts on individual’s health and major social and economic consequences in all countries of the world, it is still one of the most neglected areas of public health. In such a context, the medicinal plants traditionally used to pale these pathologies are presented as a promising tool for future drug development for the management of mental health disorders. The aim of the present study is to analyze the information about plant species used to treat mental disorders in the Catalan linguistic area (CLA) and compare these traditional uses with pharmacological literature in order to evaluate the most quoted taxa and their uses and to provide a basis for further research. **Methods:** Data have been recovered from the “Etnobotànica dels Països Catalans” webpage (https://etnobotanica.iec.cat/) and the meta-analytic work carried out in the present study covers 27 prospections performed in different territories between 1990 and 2019. Descriptive statistics and quantitative ethnobotany were carried out and some ethnobotanical indices were calculated. **Results and Discussion:** The number of use reports analysed to treat mental disorders in CLA is 2,544 spread over 183 taxa belonging to 64 families, being the most cited the Malvaceae (29.36% of use reports), Lamiaceae (16.71%), Caprifoliaceae (7.94%), Rutaceae (7.47%) and Papaveraceae (6.01%). The most used taxa to treat or alleviate the mental disorders have been *Tilia platyphyllos* Scop. (24.53%), *Valeriana officinalis* L. (7.47%), *Salvia officinalis* L. (5.07%), *Sambucus nigra* L. (4.28%), and *Ruta chalepensis* L. (3.89%). The flowers or inflorescences (47.68%), followed by aerial part (23.49%), have been the most used plant parts, and tisane the most commonly used pharmaceutical form (78.03%). The most reported use is as sedative with 40.92%, followed by anticephalalgic (21. 19%) and tranquilizer (20.01%). The informant consensus factor (FIC) was 0.93, and 3.72% was the ethnobotanicity index (EI) value. The information is coincidental with at least one of the comprehensive pharmacological literature sources checked for 73.68% of ethnobotanical uses.

## 1 Introduction

Mental health and physical health are equally important components of a holistic concept of health. The first one, sometimes underestimated, includes our emotional, psychological, and social wellbeing and it affects how we think, feel, and act in every stage of life, from childhood through adulthood (CDC, 2021). Mental disorders refer collectively to all diagnosable mental health problems, including a large range of symptoms and conditions affecting the mood, thinking and behavior, causing distress and impairment in the family, work and social areas of the individual. Although most troubles are clearly typified, the cultural context of the individual must be considered (Njoku, 2022).

According to World Health Organization (WHO) global health estimates, in 2021 more than 150 million people in the WHO European Region lived with a mental health condition (including depression, anxiety disorders and psychosis in adults, as well as developmental and behavioral disorders in children and adolescents), equivalent to 20% of the European population (WHO, 2022a).

Furthermore, mental health is interconnected with physical and social functioning, as well as health status, with a proven 10–25 years’ reduction in life expectancy for patients with severe mental disorders. Additionally, they are also attributed to a significant number of indirect deaths, since people with those pathologies have higher rates of suffering other troubles, such as type II diabetes or respiratory and cardiovascular diseases, and are more likely to commit suicide (WHO, 2013).

In addition, psychologists and mental health professionals speculate that the COVID-19 pandemic has had a further impact on the mental health of the global population, with the increase in cases of depression, suicide, and self-harm, apart from other symptoms reported globally due to the pandemic (Li et al., 2020; Moukaddam and Shah, 2020; Kumar and Nayar, 2021).

Despite these figures, mental health is one of the most neglected areas of public health, as the global median of government health expenditure that goes to mental health is less than 2% (UNICEF, 2021; WHO, 2022b), whereas the economic, social and individual burden of mental illness have clear implications in the development of the countries, with indirect costs in the labormarket, driven by lower employment rates and reduced productivity (WHO, 2018). Moreover, poor mental health was estimated to cost the world economy approximately 2.5 trillion dollars per year, a cost projected to rise to 6 trillion dollars by 2030 (The Lancet Global Health, 2020).

Despite the significant burden mental disorders impose on society, the investment and pharmaceutical innovation in this disease area remains comparatively low (MacEwan et al., 2016). In consequence, there is a need for the development of evidence-based tools and innovations for better treatments and services (WHO, 2021). In the 21st century, the pharmacological effects of medicinal plants have been considered promising for the management of healthcare (Cragg and Newman, 2013). Thereby, the key role of ethnopharmacology is to provide new approaches and novel solutions, giving to pharmaceutical companies’ supplementary knowledge about plants that can lead to innovative drugs (Heinrich and Jäger, 2015). Moreover, the benefits of plants in mental pathologies have already been reported (Parilli-Moser et al., 2021; Sarris et al., 2021; Sala-Vila et al., 2022). However, the ethnobotanical approach remains to be explored, since very few detailed monographies based on ethnobotanical studies on medicinal plants used in the treatment of mental illnesses are recorded in the literature, most of them based on African population (Romeiras et al., 2012; Ior et al., 2017; Shirungu and Cheikhyoussef, 2018; Wubetu et al., 2018; Mabaleha et al., 2019). In Europe, the ethnobotanical publications focused on mental health data are not so common (Calvo and Cavero, 2015; Motti and de Falco, 2021), but this does not mean those kinds of data are not collected during the fieldwork.

The Catalan linguistic area (CLA) is one of the most largely studied territories in Europe from the ethnobotanical point of view (Vallès, 2019), and some of the general studies show data of plants used against mental conditions (Gras et al., 2019; Gras et al., 2020), but this subject has never been addressed as a focus of the study.

Therefore, the aims of the present study are i) to analyse information about plant species used to treat mental disorders in CLA: ii) to compare these traditional uses with pharmacological literature in order to evaluate the most quoted taxa and their uses and to provide a basis for further research.

## 2 Material and methods

### 2.1 Study area

The area covered by the present study includes some territories of the CLA, which are the group of territories where Catalan is the traditional language. The Catalan linguistic area has an extension of ca. 70,000 km^2^ (Bolòs et al., 2005) and around 14,000,000 inhabitants (Departament, 2021; IBESTAT, 2021; IDESCAT, 2021; ISTAT, 2021; Portal Estadístic de la Generalitat Valenciana, 2022) belonging to four states: Andorra, France (Northern Catalonia or eastern Pyrenees department), Italy (L’Alguer, Sardinia), and Spain (Balearic Islands, Carxe—a small area in Murcia, Catalonia, a portion of eastern Aragon, and Valencia).

The wide diversity of landscape from the Mediterranean Sea level to 3,143 m a.s.l. in Pica d’Estats (Pyrenees) gives rise to a wide floristic diversity (Bolòs et al., 2005), harbouring approximately 4,300 autochthonous and 1,200 allochthonous plant taxa, including species and subspecies (Sáez, pers. comm.).

### 2.2 Databasing and data selection

The information analysed on the present paper has been collected through semi-structured ethnobotanical interviews (Pujadas et al., 2004) following the ethical principles of the International Society of Ethnobiology (ISE, 2023) and is included in the ‘Etnobotànica dels Països Catalans’ webpage (https://etnobotanica.iec.cat/) (Garnatje et al., 2021). Herbarium vouchers collected during the interviews are deposited in the herbarium BCN (Centre de Documentació de Biodiversitat Vegetal, Universitat de Barcelona). Bolòs et al.’s (2005) have been followed for taxa nomenclature, which is a flora covering specifically the area considered. In addition, Plants of the World Online (https://powo.science.kew.org) has been used for the allochtonous plants. The family attribution is performed following APG IV, the last Angiosperm Phylogeny Group’s arrangement to date (APG, 2016).

**FIGURE 1:**
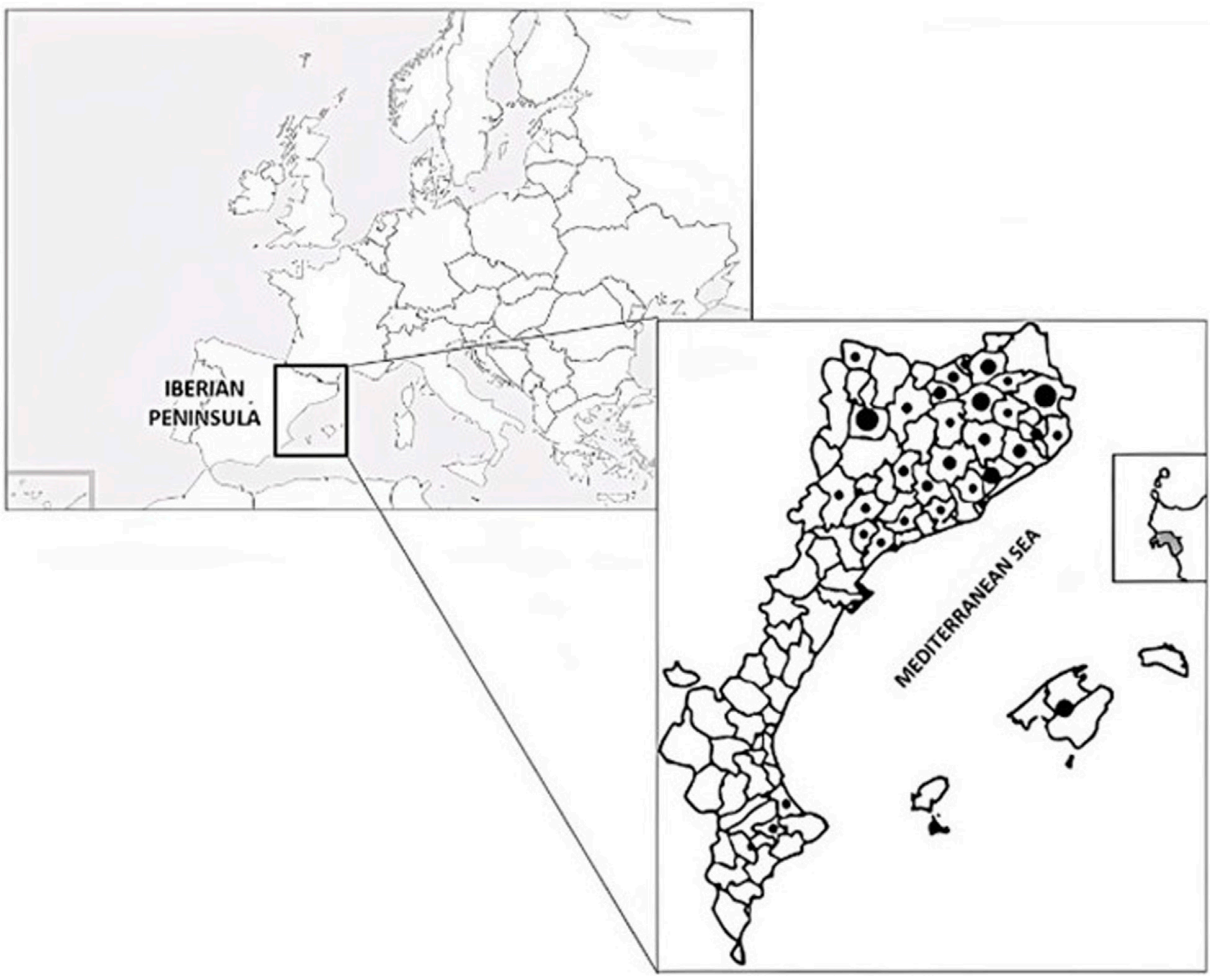
Map of the territories studied within Europe and the Catalan linguistic area. Dot size is proportional to the number of reports in each area analysed in the present study.

The information concerning mental disorders has been recovered from the mentioned database and the meta-analytic work carried out in the present study covers 27 prospections performed in different territories between 1990 and 2019 (Figure 1).

### 2.3 Data analyses

Descriptive statistics and quantitative ethnobotany were carried out using Excel (Microsoft Excel 2016), and the use report (Vandebroek et al., 2008) has been used as a unit of analysis. The relationship between the most quoted plants and their uses was visualized by an alluvial diagram using RAWGraphs (Mauri et al., 2017).

In addition, some ethnobotanical indices were calculated: 1) the informant consensus factor (F_IC_; Trotter and Logan, 1986), the ratio of the number of use reports minus the number of plant taxa used to the number of use reports minus one, in order to assess the consistency or robustness of the traditional knowledge regarding mental disorders; 2) the ethnobotanicity index (EI; Portères, 1970), the quotient between the number of plant taxa used (here taking into account the plants used for mental disorders), and the total number of plant taxa that constitute the flora of the territory (autochthonous plants, see previous estimation), expressed as a percentage, in order to have a general idea of the relevance of these plants in the area considered and to compare with the same index in studies focused on other human body systems; 3) the cultural importance index (CI; Tardío and Pardo-de-Santayana, 2008), the sum of the proportion of informants that mention each taxon use, has also been calculated to identify the most valued plants by the informants; and 4) the medicinal importance index (MI; Carrió and Vallès, 2012), the quotient between the total use reports for a specific use category and the number of plant taxa possessing this use, to evaluate the real importance of the use.

A minor bias in relation to number of use reports and related analyses exists, as in one of the studies included in the dataset (Mulet, 1990), each taxon is assigned to a municipality, instead of an informant, which is the case in all the other works.

### 2.4 Pharmacological comparison in the literature

Apart from reviewing the taxa quoted to treat mental disorders in CLA, a comparison, whenever possible, was done. The activity of the plants reported was checked in pharmacological sources as official monographs (EMA European Medicines Agency, 2022; ESCOP, 2022) and encyclopedic bibliography on phytotherapy (Blumenthal, 2003; Duke, 2003; net, 2022). The aim of this comparison, done for the twenty-five most quoted taxa, is to contrast the medicinal uses reported by the informants with pharmacological literature, thus looking for plants with potential applications, not recorded in literature, which could be of interest to develop further studies and to design new drugs.

Moreover, to correlate the medicinal uses reported with the mental disorders they are associated to, a literature search has been performed, using the two well-established systems for the classification of mental disorders: The Diagnostic and Statistical Manual of Mental Disorders IV and V, edited by the American Psychiatric Association (APA, 1994; APA, 2013); and the International Classification of Diseases by the World Health Organization (ICD, 2022).

## 3 Results and discussion

### 3.1 General data

The number of use reports collected and analysed to treat or alleviate mental disorders and associated symptoms in CLA was 2,544, and this information came from 1,082 informants. This complete dataset is available in the (Supplementary Table S1). In total, 183 taxa—eight of them determined only at genus level and 14 at infraspecific level-are quoted to treat seventeen illnesses classified as mental disorders. Taxa are distributed among 64 families and the most cited ones, representing around the 70% of total use reports, were Malvaceae (29.36%, 6 taxa), Lamiaceae (16.71%, 30 taxa), Caprifoliaceae (7.94%, 6 taxa), Rutaceae (7.47%, 5 taxa) and Papaveraceae (6.01%, 4 taxa). Unlike general ethnobotanical studies in CLA (Gras et al., 2020; Gras et al., 2021a), the most cited families are not the ones recurrently found, except for Lamiaceae, which is the second most quoted family and the most diverse in quoted taxa, followed by Asteraceae (4.91%, 18 taxa) and Rosaceae (3.66%, 10 taxa), all of them common and abundant in the Mediterranean flora (Gardner and Gardner, 2019). Moreover, the number of use reports of some of the most cited families (Malvaceae, Caprifoliaceae, Rutaceae and Papaveraceae) refers to a few taxa, meaning that there exists a large consensus on the use of these species for the treatment of mental disorders. It is worth mentioning that Malvaceae, not frequent among the top families in ethnobotanical studies, has incorporated the families Sterculiaceae and Tiliaceae and that *Tilia* is a very relevant genus for the troubles addressed (see 3.2 subheading).

The informant consensus factor (F_IC_) was 0.93. Considering that one is the maximum value for this parameter, we can state, as we mention above, that there is a high consistency of the data presented and a strong agreement among the informants for the plants used in the treatment of these illnesses in the studied area. The values for this index in some previous studies carried out in the same area were 0.92 for the treatment of infectious diseases (Gras et al., 2021b), 0.83 for respiratory tract infectious diseases (Rigat et al., 2013) and 0.93 for topical uses (Rigat et al., 2015) for local studies in more restricted areas included in CLA.

The ethnobotanicity index (EI), calculated by considering the autochthonous taxa recorded in Bolòs et al. (2005), was 3.72%. This result means that approximately 4% of the autochthonous flora is used or has been used in the past to treat mental disorders. In this case, the percentage is much lower than the calculated for the infectious diseases in the same territory (EI = 7.26%) (Gras et al., 2021a). This fact might be explained due to the self-stigma and fear of discrimination associated with such disorders, but also by the lack of information related to these pathologies, which creates a late or erroneous detection and inadequate treatment (López, 2012).

### 3.2 Most reported taxa and plant parts employed

**FIGURE 2:**
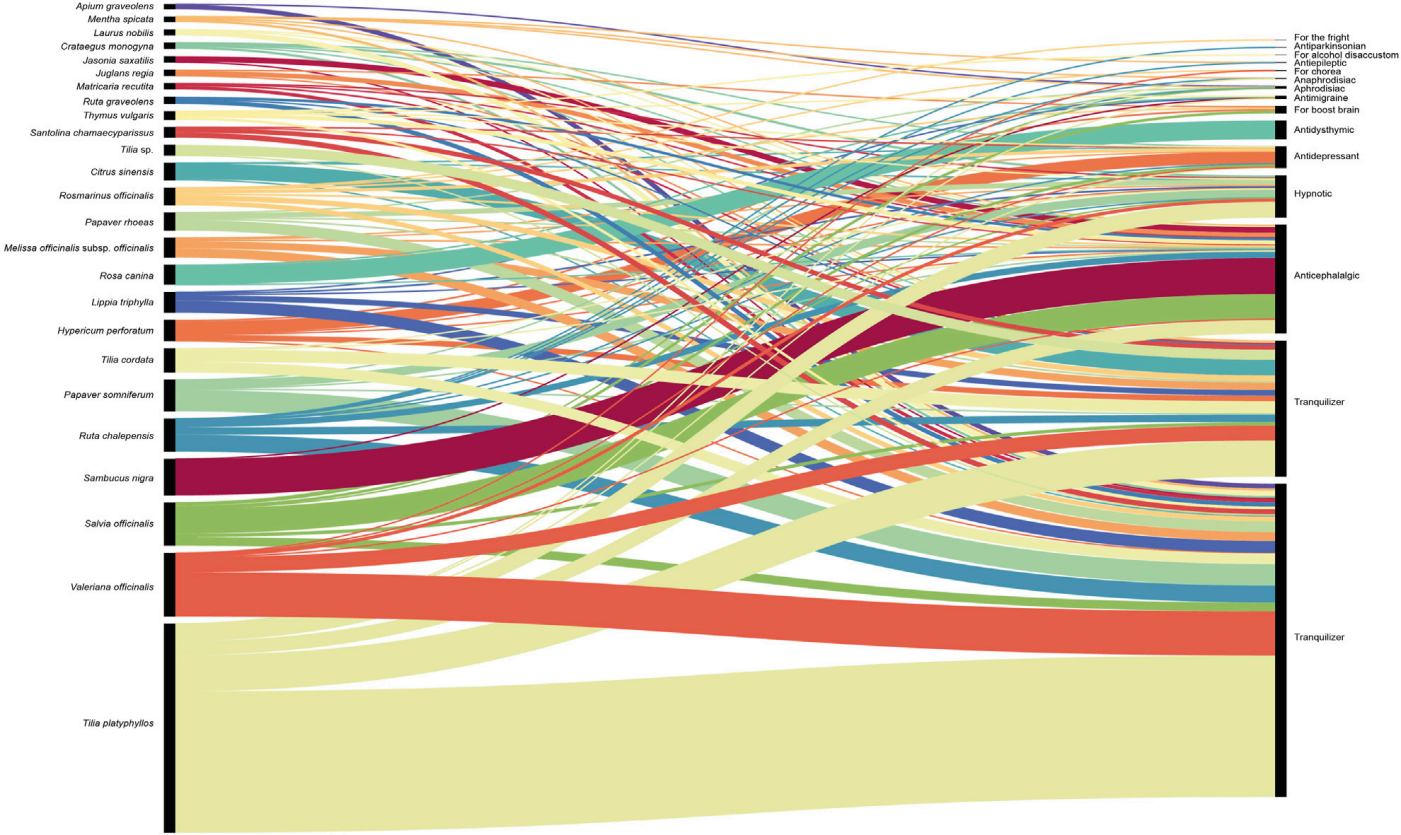
The twenty-five most cited plants and their medicinal uses quoted in the studied area.

The most quoted taxa, and their uses to treat or alleviate mental disorders in the studied areas are summarized in Supplementary Table S2 and represented in Figure 2. Among the most mentioned are *Tilia platyphyllos* Scop. (24.53%), *Valeriana. officinalis* L. (7.47%), *Salvia officinalis* L. (5.07%), *Sambucus nigra* L. (4.28%), and *Ruta. chalepensis* L. (3.89%). The genus *Tilia* is well-established in European ethnobotany to treat nervous disorders (Kozuharova et al., 2013; Jarić et al., 2014; Calvo and Cavero, 2015; Motti and de Falco, 2021) and in this study it represents 28.62% of the reports, fact that justifies the Malvaceae as the first quoted family. The roots of *V. officinalis* are also well known and established in this field (Borrás et al., 2021), and a big set of its products can be found in the market as sedative and to treat insomnia. Less frequent in the market but very quoted in this study are *S. officinalis*, *S. nigra* and *R. chalepensis*, mostly used as anticephalalgic. Cephalalgia and migraine are considered neurological disorders with a broad range of psychiatric comorbidities (APA, 2013; ICD, 2022). The most common pharmaceutical forms for these taxa and specific use are aerosol, fumigation and essences, normally not quoted as the most popular in ethnobotanical studies. The last taxon mentioned, *R. chalepensis*, is also very common among informants, mainly used as sedative and tranquilizer, taken as herbal tea or infused in chocolate, but caution should be exercised given its use as abortifacient (Gras et al., 2020; Gras et al., 2021b).

The cultural importance (CI) index was calculated for all the taxa (Supplementary Table S2). This parameter indicates the degree of agreement in plant use among informants, as it considers not only the spread of the use (number of informants for each species), reported in Supplementary Table S2, but also its versatility, i.e., the diversity of its uses, reported in Table 1. In the present study, the highest CI values are represented by *T. platyphyllos* (0.58), *V. officinalis* (0.18), *S. officinalis* (0.12), *S. nigra* (0.10) and *R. chalepensis* (0.09), and coincide with the most cited plants.

**TABLE 1:** Medicinal uses to treat mental disorders and values of total use reports (UR), total use reports percentage and medicinal importance index.

Medicinal use	Total UR	Total UR (%)	Total taxa	MI index
Sedative	1041	40.92	66	15.74
Anticephalalgic	539	21.19	74	7.26
Tranquilizer	509	20.01	61	7.77
Hypnotic	154	6.05	28	5.50
Antidysthymic	90	3.54	13	6.69
Antidepressant	82	3.22	22	3.73
For boost brain	35	1.38	14	2.50
Aphrodisiac	31	1.22	17	1.76
Antimigraine	21	0.83	13	1.62
For tobacco disaccustom	11	0.43	5	2.20
Anaphrodisiac	8	0.31	6	1.33
For alcohol disaccustom	6	0.24	4	1.50
For the fright	5	0.20	3	1.67
For onychophagy	4	0.16	1	4.00
For chorea	3	0.12	3	1.00
Antiepileptic	3	0.12	3	1.00
Antiparkinsonian	2	0,08	2	1.00

The five taxa mentioned before represent around 45% of the use reports, and the twenty-five most quoted taxa are almost 80%. Among these twenty-five taxa we can find well-known plants used in phytotherapy to treat mental disorders as *Hypericum perforatum* L. (Cervo et al., 2022), *Lippia triphylla* (L.Hér.) O.Kuntze (Bahramsoltani et al., 2018) and *Melissa officinalis* L. subsp. *officinalis* (Borrás et al., 2021), or taxa as *Santolina chamaecyparissus* L., *Jasonia saxatilis* (Lam.) Guss., and *Papaver rhoeas* L., used with the same ethnobotanical purpose in other Mediterranean areas as Navarra (Calvo and Cavero, 2015) or Italy (Motti and de Falco, 2021).

In contrast, the genus *Lavandula*, including five species growing in the CLA, and widely used for the treatment of mental disorders (Kasper, 2013; Sanei and Chasmi, 2018), has very low citation percentages in the present study (*L. dentata* L. (0.47%), *L. latifolia* Medik. (0.43%), *L. angustifolia* Mill. subsp. *pyrenaica* (DC.) Guinea (0.39%), *L. angustifolia* Mill. subsp. *angustifolia* (0.24%), *L. stoechas* L. (0.20%)).

Among the 25 most quoted taxa, *Juglans regia* L. stands out for its role on boost brain. The health-promoting benefits of this plant are ascribed to its fatty acid profile, which is rich in polyphenols and polyunsaturated fatty acids, with a particularly high ω3:ω6 ratio (Hayes et al., 2016). Moreover, previous studies have claimed that walnuts could be predicted to beneficially influence cognition (Sala-Vila et al., 2022), as both ω-3 fatty acids and polyphenols are considered critical brain nutrients (Solfrizzi et al., 2017; Scarmeas et al., 2018). The ethnobotanical approach reinforces this hypothesis, as our data show that the use of walnuts is associated to cognitive benefits for the informants of the study.

Two representatives of the genus *Papaver* (*P. somniferum* L. and *P. rhoeas*) are among the top 25 taxa in this research (Supplementary Table S2), accounting for more than 6% of all the use reports. This is in agreement with the popular and industrial uses of this genus in the field of mental disorders, coming from very old times (Bernáth, 2005). Conversely, *H. perforatum*, commonly used in folk medicine and externally applied as antalgic and anti-inflammatory (e.g., Gras et al., 2019; Gras et al., 2020), was industrially discovered as an antidepressant (Committee on herbal medicinal products, 2009), and is present in the eighth place in Supplementary Table S2, used for this purpose as well as tranquilizer and others, with roughly 2.52% of the total use reports.

Concerning the part plants used by informants to prepare remedies against mental disorders, the 96.38% (2,544 UR) refers to a known part of the plant, whereas there is no information for the remaining 3.62% of the records. The parts of the plant most commonly used to treat or alleviate mental disorders are flowers or inflorescences (47.68%) followed by aerial part (23.49%), including young, sterile, flowering and fructified aerial parts. This fact is not very surprising since all the quoted plant parts are among those more apparent and handier. This is also in accordance with other ethnobotanical studies specifically focused on mental disorders developed in Europe (Calvo and Cavero, 2015; Motti and de Falco, 2021).

### 3.3 Medicinal uses and pharmaceutical forms

The medicinal uses quoted in CLA are summarized in Table 1. The most reported use was sedative with 40.92%, followed by anticephalalgic (21.19%) and tranquilizer (20.01%). These three uses are also the highest in number of taxa, indicating a high diversity of plants used to treat mental disorders.

The medicinal importance index was calculated for all the medicinal use categories, and the results range from 1.00 to 15.74 (Table 1). The highest medicinal importance indices correspond to the most quoted uses, sedative (15.74), followed by the tranquilizer (7.77) and anticephalalgic (7.26).

The main mental conditions treated in CLA using the two well-established systems for the classifications of mental disorders (APA, 2013; ICD, 2022) are neurocognitive, anxiety, sleep, sexual behavior and depressive disorders. Therefore, plants traditionally used against the mental conditions in CLA, would have applications mainly in these kind of pathologies.

Concerning the pharmaceutical forms used, the form is indicated in 95.56% of the reports, while the remaining 4.44% has not been recorded. Among the reported forms, 89.80% are for internal use and 10.20% for external use. In total, 28 pharmaceutical forms are employed and tisane, including decoction and infusion, represents 78.03% of the total reports, followed by far by direct use (internal or external) with 6.71%. Tisane is a very common pharmaceutical form in popular medicine and the data presented in this study confirms that fact one more time (Calvo and Cavero, 2015; Gras et al., 2021b). Among the five most reported pharmaceutical forms are also fumigation (3.29%) and aerosol (2.92%).

### 3.4 Pharmacological comparison

The 25 most quoted taxa and their medicinal uses against mental disorders in CLA have been compared at the pharmacological level by reviewing official monographies and encyclopedic bibliographies on phytotherapy. Among the 114 checked uses, the information was consistent with at least one of the pharmacological sources for 73.68% ethnobotanical uses (Supplementary Table S2). By far, Duke’s CRC Handbook of Medicinal Herbs (2003) with 23 taxa and 71.05% of coinciding uses is the most inclusive and detailed work analysed, followed by net (2022) (13 taxa, 17.54%), EMA (2023) (8 taxa, 11.40%), Blumenthal (2003) (4 taxa, 5.26%) and ESCOP (2022) (3 taxa, 3.51%).

The plants quoted on ethnobotanical studies are the basis to develop traditional herbal medicinal products. The European Parliament Directive (2004/24/EC) approves that the long tradition of medicinal products simplified registration procedures and reduces the need for clinical trials, to the extendthe efficacy of the medicinal product is plausible based on long-standing use and experience (Europen Parliament, 2004). A clear example of this could be the well-known popular use of *S. nigra* or *S. officinalis* as anticephalalgic in the CLA. Focusing on the results of our study, further pharmacological investigations should be done. Furthermore, additional studies of *J. saxatilis* and *S. chamaecyparissus* uses, quoted in another Iberian Peninsula territory (Calvo and Cavero, 2015), could be interesting to explore.

## 4 Concluding remarks

The present study is the first one in the Iberian Peninsula, specifically in CLA focused on plants used to address mental disorders, and still one of the very few in Europe on this subject.

This work allows to give a perspective of the current ethnobotanical data applied to mental disorders collected so far, reflecting the data recorded since 1990 on plants traditionally used in CLA for this type of disorders. In total, a compendium of 183 taxa is quoted to treat seventeen illnesses and associated symptoms classified as mental disorders. The dataset analysed and the results presented here, demonstrate the established tradition in CLA of using plants as additional or alternative treatment to alleviate or cure the mental disorders. At the same time, the present study has assessed which of these uses are supported by pharmacological literature, showing a considerable agreement between folk and pharmacological sources, and giving a list of species that, having a great representation in the folk medicine, are not reported in official monographs. These data may be the starting point for further research with the aim to obtain alternative products to conventional treatment. Phytochemical and pharmacological studies on some of the plants quoted here would be a useful first step in this process.

The importance of ethnobotany should also be emphasized because, as shown here, this science has a great potential to become a key step in drug development. Moreover, in a world in which the optimization of all systems is sought, it is important to look at the past from the present in order to face the future with new interpretations. In this line, ethnobotany is necessary to rescue these uses and rely on them for the development of new drugs and applications.

## Data Availability

The original contributions presented in the study are included in the article/Supplementary Material, further inquiries can be directed to the corresponding author.

## Supplementary material

SUPPLEMENTARY TABLE S1 The twenty-five most cited plants to alleviate or treat mental disorders in the studied area, with the medicinal uses, the number of total use reports (UR) and percentage and the Cultural Importance (CI) index. Comparison of uses in pharmacological comprehensive literature: (1) EMA (2022), (2) ESCOP (2022), (3) Fitoterapia.net (2022), (4) Duke (2003), (5) Blumenthal (2003).

## Abbreviations

CI, Cultural Importance Index; CLA, Catalan linguistic area; EI, Ethnobotanicity Index; EMA, European Medicine Agency; ESCOP, European Scientific Cooperative on Phytotherapy; F_IC_, Informant Consensus Factor; MI index, Medicinal Importance index; UR, Use Reports; WHO, World Health Organization.

## References

[B1] APA (1994). Diagnostic and statistical manual of mental disorders. 4th ed. Washington DC: American Psychiatric Publishing, Inc.

[B2] APA (2013). Diagnostic and statistical manual of mental disorders. 5th ed. Washington DC: American Psychiatric Publishing, Inc.

[B3] APG (2016). An update of the angiosperm phylogeny group classification for the orders and families of flowering plants: APG IV. Bot. J. Linn. Soc. 181, 1–20. 10.1111/boj.12385

[B4] Bahramsoltani R. Rostamiasrabadi P. Shahpiri Z. Marques A. M. Rahimi R. Farzaei M. H. (2018). AloysiacitrodoraPaláu (Lemon verbena): a review of phytochemistry and pharmacology. J. Ethnopharmacol. 222, 34–51. 10.1016/j.jep.2018.04.021 29698776

[B5] Bernáth J. (Editor) (2005). *Poppy. The genus* Papaver*. Medicinal and aromatic plants – Industrial profiles* (Amsterdam: Harwood Academic Publishers).

[B6] Blumenthal M. (2003). The ABC clinical guide to herbs. Austin: American Botanical Council.

[B7] Bolòs O. de. Vigo J. Masalles R. M. Ninot J. (2005). Flora manual dels Països Catalans. 3rd ed. Barcelona: Editorial Pòrtic.

[B8] Borrás S. Martínez-Solís I. Ríos J. L. (2021). Medicinal plants for insomnia related to anxiety: an updated review. Planta Med. 87, 738–753. 10.1055/a-1510-9826 34116572

[B9] Calvo M. I. Cavero R. Y. (2015). Medicinal plants used for neurological and mental disorders in Navarra and their validation from official sources. J. Ethnopharmacol. 169, 263–268. 10.1016/j.jep.2015.04.035 25922267

[B10] Carrió E. Vallès J. (2012). Ethnobotany of medicinal plants used in eastern Mallorca (Balearic Islands, Mediterranean Sea). J. Ethnopharmacol. 141, 1021–1040. 10.1016/j.jep.2012.03.049 22783553

[B11] CDC (2021). About mental health. Available at: https://www.cdc.gov/mentalhealth/learn/index.htm (Accessed February 21, 2023).

[B12] Cervo L. Rozio M. Ekalle-Soppo C. Guiso G. Morazzoni P. Caccia S. (2002). Role of hyperforin in the antidepressant-like activity of *Hypericum perforatum* extracts. Psychopharmacology 164 (4), 423–428. 10.1007/s00213-002-1229-5 12457273

[B13] Committee on herbal medicinal products (2019). Assessment report on *Hypericum perforatum* L. herba. London: European Medicines Agency.

[B14] Cragg G. M. Newman D. J. (2013). Natural products: a continuing source of novel drug leads. Biophys. Acta Gen. Subj. 1830 (6), 3670–3695. 10.1016/j.bbagen.2013.02.008 PMC3672862 23428572

[B15] Departament (2021). Departament d’Estadística del Govern d’Andorra. Available at: https://www.estadistica.ad/.

[B16] Duke J. A. (2003). CRC Handbook of medicinal herbs. 2nd ed. Austin: American Botanical Council.

[B17] EMA European Medicines Agency (2022). European Union herbal monographs. Available at: https://www.ema.europa.eu/en.

[B18] ESCOP (2022). ESCOP monographs: the Scientific Foundation for herbal medicinal products. Available at: https://escop.com/.

[B19] European Parliament (2004). Directive 2004/24/EC of the European Parliament and of the Council of 31 March 2004 amending, as regards traditional herbal medicinal products, Directive 2001/83/EC on the Community code relating to medicinal products for human use. Official J. Eur. Union L136, 85–90.

[B74] Fitoterapia.net (2022). Vademécum de Fitoterapia. Available at: https://www.fitoterapia.net/ .

[B20] Gardner C. Gardner B. (2019). Flora of the Mediterranean: an illustrated guide. London: Bloomsbury Publishing.

[B21] Garnatje T. Gras A. Parada J. Parada M. Vallès J. (2021). La web ‘Etnobotànica dels Països Catalans’: coneixement tradicional al servei de la societat. Coll. Bot. 40, e006. 10.3989/collectbot.2021.v40.006

[B22] Gras A. Hidalgo O. D’Ambrosio U. Parada M. Garnatje T. Valles J. (2021a). The role of botanical families in medicinal ethnobotany: a phylogenetic perspective. Plants 10 (1), 163. 10.3390/plants10010163 33467763 PMC7830233

[B23] Gras A. Parada M. Vallès J. Garnatje T. (2021b). The role of traditional plant knowledge in the fight against infectious diseases: a meta-analytic study in the Catalan linguistic area. Front. Pharmacol. 12, 744616. 10.3389/fphar.2021.744616 34707501 PMC8543157

[B24] Gras A. Serrasolses G. Vallès J. Garnatje T. (2019). Traditional knowledge in semi-rural close to industrial areas: ethnobotanical studies in western Gironès (Catalonia, Iberian Peninsula). J. Ethnobiol. Ethnomed. 15 (1), 19. 10.1186/s13002-019-0295-2 30940210 PMC6444684

[B25] Gras A. Vallès J. Garnatje T. (2020). Filling the gaps: ethnobotanical study of the Garrigues district, an arid zone in Catalonia (NE Iberian Peninsula). Ethnobiol. Ethnomed. 16 (1), 34–15. 10.1186/s13002-020-00386-0 PMC7285587 32517701

[B26] Hayes D. Angove M. J. Tucci J. Dennis C. (2016). Walnuts (*Juglans regia*) chemical composition and research in human health. Crit. Rev. Food Sci. Nutr. 56 (8), 1231–1241. 10.1080/10408398.2012.760516 25747270

[B27] Heinrich M. Jäger A. K. (2015). Ethnopharmacology. Chichester: John Wiley & Sons.

[B28] IBESTAT (2021). IBESTAT (Institut d’Estadística de les Illes Balears. Available at: https://ibestat.caib.es/ibestat/inici (Accessed June 5, 2023).

[B29] ICD. (2022). International classification of diseases 11th Revision. The global standard for diagnostic health information. World Health Organization. Available at: https://icd.who.int/en (Accessed June 15, 2023).

[B30] IDESCAT (2021). IDESCAT (Institut d’Estadística de Catalunya). Available at: https://www.idescat.cat/ (Accessed June 5, 2023).

[B31] Ior L. D. Otimenyin S. O. Okwori V. A. Umar D. M. Azila J. J. (2017). Ethnobotanical survey of plants used in the management of mental illnesses in some selected local government areas of Plateau State, Nigeria. J. Pharmacogn. 9 (10), 146–156. 10.5897/JPP2017.0464

[B32] ISE (International Society of Ethnobiology) (2023). International society of Ethnobiology code of Ethics. (with 2008 additions). Available at: http://ethnobiology.net/code-of-ethics (Accessed January 30, 2023).

[B33] ISTAT (2021). ISTAT (Istituto Nazionale di Statistica). Available at: https://www.istat.it/.

[B34] Jarić S. Mitrović M. Pavlović P. (2014). “An ethnobotanical and ethnomedicinal study on the use of wild medicinal plants in rural areas of Serbia,” in Ethnobotany and biocultural diversities in the Balkans. Editors Pieroni A. Quave C. L. (New York: Springer), 87–112.

[B35] Kasper S. (2013). An orally administered lavandula oil preparation (Silexan) for anxiety disorder and related conditions: an evidence based review. Int. J. Psychiatry Clin. Pract. 17, 15–22. 10.3109/13651501.2013.813555 23808618

[B36] Kozuharova E. Lebanova H. Getov I. Benbassat N. Napier J. (2013). Descriptive study of contemporary status of the traditional knowledge on medicinal plants in Bulgaria. Afr. J. Pharm. Pharmacol. 7 (5), 185–198. 10.5897/AJPP12.871

[B37] Kumar A. Nayar K. R. (2021). COVID 19 and its mental health consequences. J. Ment. Health 30 (1), 1–2. 10.1080/09638237.2020.1757052 32339041

[B38] Li Z. Ge J. Yang M. Feng J. Qiao M. Jiang R. (2020). Vicarious traumatization in the general public, members, and non-members of medical teams aiding in COVID-19 control. Brain Behav. Immun. 88, 916–919. 10.1016/j.bbi.2020.03.007 32169498 PMC7102670

[B39] López M. (2012). El estigma en salud mental. Psychol. Soc. Educ. 4 (2), 131–136. 10.25115/psye.v4i2.486

[B40] Mabaleha M. B. Zietsman P. C. Wilhelm A. Bonnet S. L. (2019). Ethnobotanical survey of medicinal plants used to treat mental illnesses in the Berea, Leribe, and Maseru Districts of Lesotho. Nat. Prod. Com. 14 (7), 1934578X1986421. 10.1177/1934578x19864215

[B41] MacEwan J. P. Seabury S. Aigbogun M. S. Kamat S. van Eijndhoven E. Francois C. (2016). Pharmaceutical innovation in the treatment of schizophrenia and mental disorders compared with other diseases. Innov. Clin. Neurosci. 13 (7-8), 17–25. 27672484 PMC5022985

[B42] Mauri M. Elli T. Caviglia G. Uboldi G. Azzi M. (2017).RAWGraphs, Proceedings of the 12th Biannual Conference on Italian SIGCHI, Vol. 28. New York: Chapter-CHItaly 17, 28.

[B43] Motti R. de Falco B. (2021). Traditional herbal remedies used for managing anxiety and insomnia in Italy: an ethnopharmacological overview. Horticulturae 7 (12), 523. 10.3390/horticulturae7120523

[B44] Moukaddam N. Shah A. (2020). Psychiatrists beware! The impact of COVID-19 and pandemics on mental health. Available at: https://www.psychiatrictimes.com/view/psychiatrists-beware-impact-coronavirus-pandemics-mental-health . Psychiatr. Times 37 (3).

[B45] Mulet L. (1990). Aportaciones al conocimiento etnobotánico de la provincia de Castellón. [PhD Thesis]. València: Universitat de València.

[B46] net (2022). Vademécum de Fitoterapia. Available at: https://www.fitoterapia.net/.

[B47] Njoku I. (2022). What is mental Illness? Available at: https://www.psychiatry.org/patients-families/what-is-mental-illness (Accessed November 7, 2023).

[B48] Our World in Data (2021). Mental health. Available at: https://ourworldindata.org/mental-health (Accessed February 24, 2023).

[B49] Parilli-Moser I. Domínguez-López I. Trius-Soler M. Castellví M. Bosch B. Castro-Barquero S. (2021). Consumption of peanut products improves memory and stress response in healthy adults from the ARISTOTLE study: a 6-month randomized controlled trial. Clin. Nutr. 40 (11), 5556–5567. 10.1016/j.clnu.2021.09.020 34656952

[B50] Portal Estadístic de la Generalitat Valenciana (2022). Available at: https://pegv.gva.es/va/.

[B51] Portères R. (1970). Ethno-botanique générale. Paris: Muséum National d’Histoire Naturelle/Faculté des Lettres.

[B52] Pujadas J. J. Comas D. Roca J. (2004). Etnografia. Barcelona: Universitat Oberta de Catalunya.

[B53] Rigat M. Vallès J. Gras A. Iglésias J. Garnatje T. (2015). Plants with topical uses in the Ripollès district (Pyrenees, Catalonia, Iberian Peninsula): ethnobotanical survey and pharmacological validation in the literature. J. Ethnopharmacol. 164, 162–179. 10.1016/j.jep.2015.01.055 25666424

[B54] Rigat M. Vallès J. Iglésias J. Garnatje T. (2013). Traditional and alternative natural therapeutic products used in the treatment of respiratory tract Infectious diseases in the eastern Catalan Pyrenees (Iberian Peninsula). J. Ethnopharmacol. 148 (2), 411–422. 10.1016/j.jep.2013.04.022 23612419

[B55] Romeiras M. M. Duarte M. C. Indjai B. Catarino L. (2012). Medicinal plants used to treat neurological disorders in West Africa: a case study with Guinea-Bissau flora. Am. J. Plant Sci. 3 (7A), 1028–1036. 10.4236/ajps.2012.327122

[B56] Sala-Vila A. Fleming J. Kris-Etherton P. Ros E. (2022). Impact of α-linolenic acid, the vegetable ω-3 fatty acid, on cardiovascular disease and cognition. Adv. Nutr. 13 (5), 1584–1602. 10.1093/advances/nmac016 35170723 PMC9526859

[B57] Sanei R. Chasmi F. N. (2018). Effect of Lavandula angustifolia extract to prevent test anxiety (A study on students at two time intervals). Entomol. Appl. Sci. Let. 5 (1), 1–6.

[B58] Sarris J. Wolfgang M. Ashton M. M. Ng C. H. Galvao-Coelho N. Ayati Z. (2021). Plant-based medicines (Phytoceuticals) in the treatment of psychiatric disorders: a meta-review of meta-analyses of randomized controlled trials. Les médicaments à base de plantes (phytoceutiques) dans le traitement des troubles psychiatriques: une méta-revue des méta-analyses d’essais randomisés contrôlés. Can. J. Psychiatry. 66 (10), 849–862. 10.1177/0706743720979917 33596697 PMC8573706

[B59] Scarmeas N. Anastasiou C. A. Yannakoulia M. (2018). Nutrition and prevention of cognitive impairment. Lancet Neurol. 17 (11), 1006–1015. 10.1016/S1474-4422(18)30338-7 30244829

[B60] Shirungu M. M. Cheikhyoussef A. (2018). Therapeutic powers of medicinal plants used by traditional healers in Kavango, Namibia, for mental illness. Anthropol. South. Afr. 41 (2), 127–135. 10.1080/23323256.2018.1486217

[B61] Solfrizzi V. Custodero C. Lozupone M. Imbimbo B. P. Valiani V. Agosti P. (2017). Relationships of dietary patterns, foods, and micro- and macronutrients with Alzheimer’s disease and late-life cognitive disorders: a systematic review. J. Alzheimer’s Dis. 59, 815–849. 10.3233/JAD-170248 28697569

[B62] Tardío J. Pardo-de-Santayana M. (2008). Cultural importance indices: a comparative analysis based on the useful wild plants of southern Cantabria (northern Spain). Econ. Bot. 62, 24–39. 10.1007/s12231-007-9004-5

[B63] The Lancet Global Health (2020). Mental health matters. Lancet 8 (11), E1352. 10.1016/S2214-109X(20)30432-0 PMC7561290 33069297

[B64] Trotter R. T. Logan M. H. (1986). “Informant Consensus: a New Approach for identifying potentially effective medicinal plants,” in Plants in indigenous medicine and diet, behavioral approaches. Editor Etkin N. L. (New York: Redgrave Publishing Company), 91–112.

[B65] UNICEF (2021). The state of the world’s children 2021. Available at: https://www.unicef.org/reports/state-worlds-children-2021 (Accessed July 10, 2023).

[B66] Vallès J. (2019). Etnobotànica: persones, plantes, cultura i benestar: aspectos generals, i situació i perspectives als Països Catalans. Barcelona: Institut d'Estudis Catalans.

[B67] Vandebroek I. Thomas E. Sanca S. Van Damme P. Puyvelde L. V. De Kimpe N. (2008). Comparison of health conditions treated with traditional and biomedical health care in a Quechua community in rural Bolivia. J. Ethnobiol. Ethnomed. 4, 1. 10.1186/1746-4269-4-1 18194568 PMC2265262

[B68] WHO (World Health Organization) (2022a). European framework for action on mental health 2021–2025. Copenhagen: WHO Regional Office for Europe.

[B69] WHO (World Health Organization) (2018). International classification of diseases for mortality and morbidity statistics (11th Revision). Available at: https://icd.who.int/browse11/l-m/en (Accessed June 7, 2023).

[B70] WHO (World Health Organization) (2013). Premature death among people with severe mental disorders. Available at: https://www.who.int/mental_health/management/info_sheet.pdf (Accessed June 7, 2023).

[B71] WHO (World Health Organization) (2021). WHO European framework for action on mental health 2021–2025. Draft for the Seventy-first Regional Committee for Europe. Copenhagen: WHO Regional Office for Europe.

[B72] WHO (World Health Organization) (2022b). World mental health report: transforming mental health for all. Geneva: World Health Organization.

[B73] Wubetu M. Sintayehu M. Aeta M. A. (2018). Ethnobotany of medicinal plants used to treat various mental illnesses in Ethiopia: a systematic review. Asian J. Plant Sci. 8 (1), 9–33.

